# Development of a Cantilever-Type Electrostatic Energy Harvester and Its Charging Characteristics on a Highway Viaduct

**DOI:** 10.3390/mi8100293

**Published:** 2017-09-28

**Authors:** Hideaki Koga, Hiroyuki Mitsuya, Hiroaki Honma, Hiroyuki Fujita, Hiroshi Toshiyoshi, Gen Hashiguchi

**Affiliations:** 1 Saginomiya Seisakusho, Inc., 535 Sasai, Sayama, Saitama 350-1395, Japan; hiro-mitsuya@saginomiya.co.jp; 2 Institute of Industrial Science, The University of Tokyo, 4-6-1 Komaba, Meguro-ku, Tokyo 153-8505, Japan; honma-hh@iis.u-tokyo.ac.jp (H.H.); hfujita@iis.u-tokyo.ac.jp (H.F.); hiro@iis.u-tokyo.ac.jp (H.T.); 3 Department of Mechanical Engineering, Shizuoka University, 3-5-1 Jyouhoku, Naka-ku, Hamamatsu, Shizuoka 432-8011, Japan; hashiguchi.gen@shizuoka.ac.jp

**Keywords:** electrostatic energy harvester, electret, potassium ion electret, comb-drive

## Abstract

We have developed a micro-electro-mechanical systems (MEMS) electrostatic vibratory power generator with over 100 μW${}_{\mathrm{RMS}}$ of (root-mean-square) output electric power under 0.03 G${}_{\mathrm{RMS}}$ (G: the acceleration of gravity) accelerations. The device is made of a silicon-on-insulator (SOI) wafer and is fabricated by silicon micromachining technology. An electret built-in potential is given to the device by electrothermal polarization in silicon oxide using potassium ions. The force factor, which is defined by a proportional coefficient of the output current with respect to the vibration velocity, is 2.34 × 10${}^{-4}$ C/m; this large value allows the developed vibration power generator to have a very high power efficiency of 80.7%. We have also demonstrated a charging experiment by using an environmental acceleration waveform with an average amplitude of about 0.03 G${}_{\mathrm{RMS}}$ taken at a viaduct of a highway, and we obtained 4.8 mJ of electric energy stored in a 44 μF capacitor in 90 min.

## 1. Introduction

Recently, an increasing number of studies have been performed on micro-electro-mechanical systems (MEMS) vibration power generators to realize autonomous electric power sources for sensor network systems. Energy harvesters are categorized into three types depending on the operational principles, that is, electromagnetic transduction (EM) [1,2], electrostatic transduction (ES) [1,3], or piezoelectric transduction (PZ) [1,4]. Triboelectric transduction [5,6,7] is one of the promising principles for autonomous power generation, but this involves direct electric carrier transport via the physical contact of materials rather than the energy exchange of “fields”; hence, we exclude it from the scope of the introduction.

Good reviews of vibration power generators for EM, ES, and PZ devices have been given by Beeby et al. in 2006 [8] and by Mitcheson et al. in 2008 [9]. In these reviews, the characteristics of vibration energy harvesting devices are summarized by the same categories. The history of MEMS energy harvesters shows a trend of an increasing weight of the proof mass for a larger output power. For example, a 6 μW output was reported on an ES power generator with a 0.65 g proof mass [10], while Despesse et al. have shown 1.76 mW on a 104 g mass in an ES harvester [11]. Using a PZ harvester, 2.1 μW was retrieved with a 0.8 g proof mass [4], while 375 μW was obtained from a 9.2 g mass [12]. Furthermore, Saha et al. have demonstrated 3.2 mW of output power on a 25 g proof mass by an EM harvester [2]. Because the kinetic energy of mechanical vibration is proportional to the proof mass, an increase in mass is the first strategy to be considered to obtain high electric power from vibrations.

Research on vibration energy harvesters has continued with keen interest and has been widely reported in literature. The focus of the many issues seems to be the power conversion efficiency and wide-frequency response of vibration power generators, in anticipation of practical use. For example, Wei et al. have reported a 25% energy conversion efficiency by using PZ devices made of piezoelectric lead zirconate titanate (PZT) film, and they showed the effects on device parameters for cantilever vibration. According to the parameter optimization, they have established 120 μW${}_{\mathrm{RMS}}$ output power under a 1 G${}_{\mathrm{RMS}}$ sinusoidal acceleration [13]. Tang and Zuo analyzed a dual-mass configuration to enhance the output energy and showed that it would enhance the average power compared with a single-mass system [14]. Karami et al. conducted a parametric study for PZ devices both analytically and experimentally, and suggested that there is an optimum substrate-to-piezoelectric thickness ratio, depending on the substrate materials [15]. Basari et al. investigated the shape effect of cantilevers for a piezoelectric energy harvester and found that the power efficiency of the triangular plate cantilever is higher than that of the rectangular plate [16]. Jia and Seshia demonstrated that a cantilever-type vibration energy harvester with an end-mass occupying about 60% to 70% of the total cantilever length outperforms comparable devices with 40% and 50% ratios [17]. A recent review on PZT-based energy harvesters has been reported by M. G. Kang et al. [18]. While many studies have been performed on PZ devices, studies of ES devices that employed electret film have also drawn attention to improve device performance [19,20,21,22]. Y. Suzuki gave a comprehensive review in 2011, in which the details of electric power generation using the electret technique and progress up to that point are reported [3]. A more recent study conducted by M. Renaud et al. established over 160 μW of output power by an ES device with 2.9 G acceleration [23]. As a result of the efforts conducted by many researchers, vibratory energy harvesters are now close to a practical level.

However, we seem to have a lack of understanding of relations between device parameters and power conversion efficiency, and we hence have not yet reached the full potential of vibration power generators. Furthermore, the acceleration of environmental vibrations observed on infrastructures is very small. For example, a RMS value we measured at a viaduct for a highway was about 0.03 G${}_{\mathrm{RMS}}$, and therefore we need to develop a vibration power generator that enables us to charge sufficient electric energy to operate a sensor network device from such a small vibration source. To our best knowledge, there are few papers that report electrically charged energy retrieved from environmental vibrations.

In this paper, we demonstrate a new MEMS electrostatic power generator utilizing potassium ion electrets. The developed device with a 14.4 g proof mass exhibited 115 μW${}_{RMS}$ power generation by 0.03 G${}_{\mathrm{RMS}}$ acceleration. Using the developed power generator, we conducted a charging experiment under an acceleration waveform observed on a viaduct and established an electric energy of 4.8 mJ stored in a 44 μF capacitor in 90 min.

## 2. Device Modeling and Design Parameters

**Figure 1:**
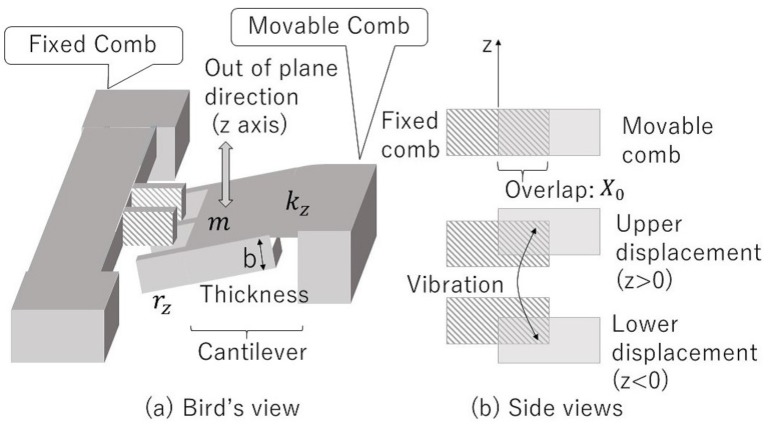
(**a**) A schematic model of the vibration energy harvester. Comb-drive electrodes are formed at a cantilever structure that vibrates in the out-of-plane direction; (**b**) A side-view image of relative positions between the fixed and movable comb-drive electrodes.

**Figure 2:**
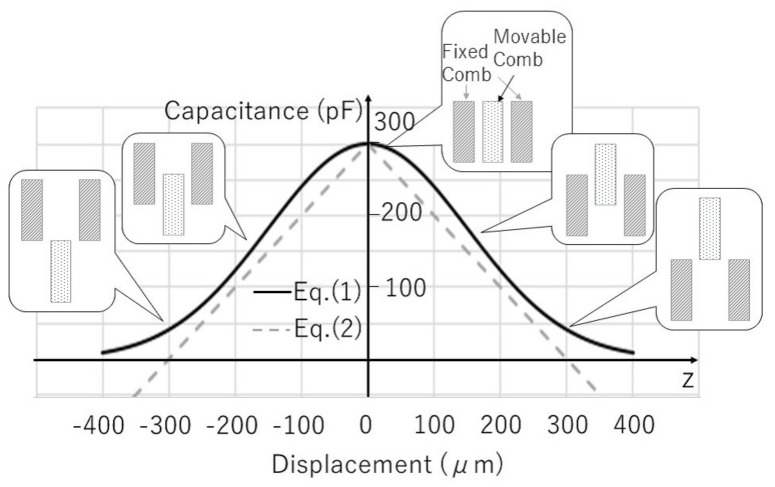
Comparison of the capacitance functions. The fitting parameter $z_{0}$ for the Gaussian function form is chosen to be 150 μm for b=300μm.

The device model we have investigated and fabricated in this study is illustrated in Figure 1. Comb electrodes are formed at the tip of a cantilever structure with a spring constant *k*${}_{z}$ that vibrates in the out-of-plane direction (z-direction). Because both the upper and lower movements of the movable comb electrode equally cause a decrease of the overlapping area between the comb electrodes, the capacitance should be represented by an even function of the displacement *z*. Therefore in this work, we modeled the capacitance by a Gaussian function form as

(1) $$\begin{array}{c} C\left(z\right)=C_{0}\exp\left(-\frac{z^{2}}{z_{0}^{2}}\right) \end{array}$$

where *C*${}_{0}$ is the capacitance at *z* = 0 (initial position), and *z*${}_{0}$ is a fitting parameter. The cantilever makes a rotational motion at the tip, but its behavior is described by a vertical motion because the cantilever length is considerably longer than the thickness of the comb electrodes. Therefore, if the overlapping area lineally changes with the displacement, the capacitance function is represented by

(2) $$\begin{array}{c} C\left(z\right)=\frac{2n\epsilon_{0}X_{0}}{d}\left(b-\left|z\right|\right) \end{array}$$

where *n* is the number of the comb electrode pairs, $\epsilon_{0}$ is the permittivity of vacuum, *X*${}_{0}$ is the designed overlap in the direction of the comb length, and *b* and *d* are the thickness of the comb electrodes and the gap between the fixed and the movable comb electrodes, respectively. Any parasitic capacitance is not considered. Figure 2 shows a comparison of the capacitances calculated by the above two equations as a function of the displacement *z*. We use the designed values listed in Table 1.

The Gaussian form guarantees continuity of the capacitance and it seems to represent the true capacitance dependency, especially in the case of a large displacement, because the capacitance formed by the opposing comb electrodes should diminish when the movable comb electrode moves far from the fixed comb electrode. Furthermore, considering the fringe electric fields between the opposing electrodes, the capacitance value will be larger than the ideal case of Equation (2), and the capacitance change is rounded around z=0 because of the contribution of the fringe electric field at the top and the bottom surfaces of the comb electrodes. The Gaussian form well represents these matters; hence, we adapt the Gaussian function for modeling.

The Lagrange function of the device shown in Figure 1 is thus written in the following form:

(3) $$\begin{array}{c} Ł=\frac{1}{2}mv^{2}+\frac{1}{2}C\left(z\right){\left(E+e\right)}^{2}-\frac{1}{2}k_{z}z^{2} \end{array}$$

where *v* is the vibration velocity of the movable comb electrode; *m* the effective mass as a lumped value at the tip of the cantilever, including the proof mass; and *E* and *e*, which are defined as the generalized velocity in the Lagrangian, are the bias voltage and the alternating voltage generated between two comb electrodes, respectively. The dissipation function of the device is also given by

(4) $$\begin{array}{c} \Gamma =\frac{1}{2}rv^{2} \end{array}$$

where *r* is the damping factor.

The motion equations of this device are then derived as follows [24]:

(5) $$\begin{array}{cc} f & =\frac{d}{dt}\left(\frac{\partial Ł}{\partial v}\right)-\frac{\partial Ł}{\partial z}+\frac{\partial \Gamma }{\partial v}\\ & =m\frac{dv}{dt}+rv+kz+\frac{C_{0}}{z_{0}^{2}}e^{2}z\exp\left(-\frac{z^{2}}{z_{0}^{2}}\right) \end{array}$$

(6) $$\begin{array}{ccc} i & = & \frac{d}{dt}\left(\frac{\partial Ł}{\partial e}\right)=-\frac{2C_{0}}{z_{0}^{2}}evz\exp\left(-\frac{z^{2}}{z_{0}^{2}}\right)+C\left(z\right)\frac{de}{dt} \end{array}$$

where *f* is the external force applied to the effective mass, and *i* is the output current of the device. From Equation (5), the displacement of the cantilever at the static balance, *z*${}_{s}$, made by the gravitational force of the effective mass is determined by solving the following equation:

(7) $$\begin{array}{c} mg=kz_{s}+\frac{z_{s}E^{2}}{z_{0}^{2}}C_{0}\exp\left(-\frac{z_{s}^{2}}{z_{0}^{2}}\right) \end{array}$$

where *G* is the acceleration of gravity (*G* = 9.8 m/s${}^{2}$) and *E* is the applied electret potential. The linear motion equations around the static point $z=z_{s}$ are then obtained and written as

(8) $$\begin{array}{ccc} f & = & m\frac{dv}{dt}+rv+k^{\prime }z+Ae \end{array}$$

(9) $$\begin{array}{ccc} i & = & -Av+C_{s}\frac{de}{dt} \end{array}$$

(10) $$\begin{array}{ccc} k^{\prime } & = & k+\frac{C_{s}E^{2}}{z_{0}^{2}}\left(1-\frac{2z_{s}^{2}}{z_{0}^{2}}\right) \end{array}$$

(11) $$\begin{array}{ccc} A & = & \frac{2z_{s}C_{s}E}{z_{0}^{2}} \end{array}$$

(12) $$\begin{array}{ccc} C_{s} & = & C_{0}\exp\left(-z_{s}^{2}/z_{0}^{2}\right) \end{array}$$

The parameter *A* is not only the conversion factor of voltage into mechanical force, but it is also of velocity into current. However, this parameter is only related to the power efficiency of the vibration power generator. Presuming that the same impedance as the internal impedance of the vibration power generator is connected to the device, the expected RMS output power is given as follows [24]:

(13) $$\begin{array}{c} P=\frac{ma^{2}Q}{4\omega_{0}}\cdot \frac{1}{1+{\left(\frac{k^{\prime }C_{s}}{QA^{2}}\right)}^{2}} \end{array}$$

where *a* is the RMS acceleration value, *Q* is the quality factor of the resonance vibration under no-load conditions, and $\omega_{0}$ is the resonance angular velocity. The first fraction of the product shows the maximum output power, which is only determined by the mechanical parameters and which corresponds to its ideal output retrieved from the mechanical input power (force × vibration velocity; fv), while the second fraction of the product corresponds to the harvester effectiveness for the possible maximum output power, giving

(14) $$\begin{array}{c} \eta =\frac{P}{P_{max}}=\frac{1}{1+{\left(\frac{k^{\prime }C_{s}}{QA^{2}}\right)}^{2}} \end{array}$$

Clearly, this expresses the ratio of the obtained power with respect to the maximum theoretical available power [9]. In order to establish a high-efficiency power generator, large *A* and low $k^{\prime }$ values are required. The $k^{\prime }$ value is the effective spring constant, which includes an equivalent spring effect yielded by the electrostatic force. From Equation (10), the effective spring constant changes to be softer than the mechanical spring constant *k* when $z_{s}$ exceeds $z_{0}/\sqrt{2}$; this is advantageous for designing a vibration power generator with a low resonant frequency. The design parameters of the cantilever-type vibration power generator are listed in Table 1. Using these parameters, $C_{0}$ becomes about 300 pF, without considering the fringe electric fields between two comb electrodes. The expected force factor in this design is over 2 × 10${}^{-4}$ C/m, which is enough to establish 100% power efficiency when the *Q* value is ensured to be over 50.

## 3. Fabrication of the Vibration Energy Harvester

**Figure 3:**
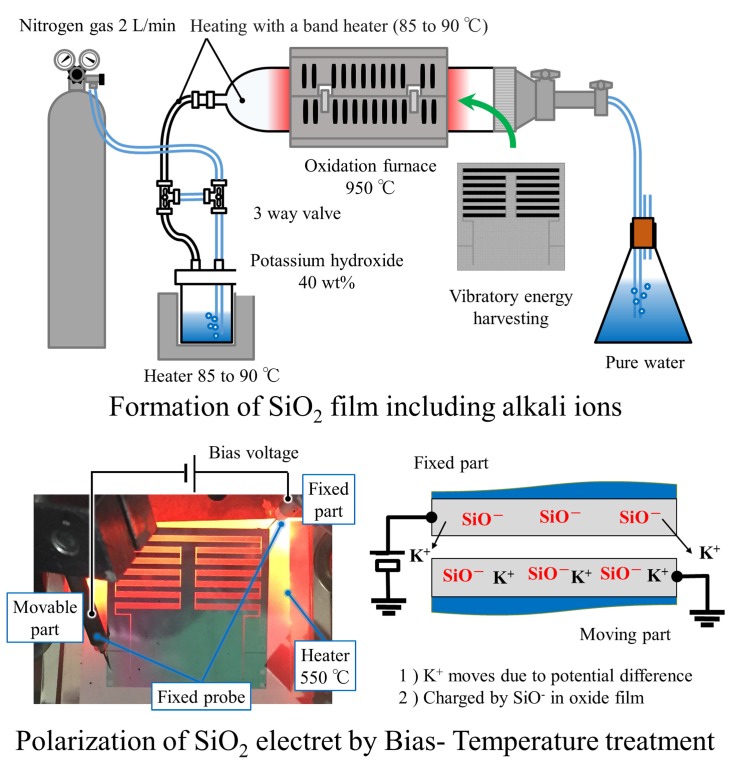
Formation of SiO${}_{2}$ electret.

We fabricated the out-of-plane vibration device as an energy harvester using conventional microfabrication techniques and the potassium ion electret technique [25]. The device is made of a silicon-on-insulator (SOI) wafer with a 300 μm thick device layer, a 3 μm thick buried oxide layer, and a 450 μm thick substrate. First, a silicon nitride film was deposited on the SOI wafer and patterned to cover the electric pad area by photolithography and reactive ion etching processes. Then the device structure was formed by etching the SOI layer by photolithography and deep trench etching technology. In order to release the movable parts of the device from the substrate, photolithography and deep trench etching were utilized again to etch the substrate from the backside of the SOI wafer. After removing the exposed buried oxide layer, the device was oxidized at a temperature of 950 °C by a bubbled stream through a warmed KOH solution. By this process, the device was covered with an oxide film that incorporated potassium ions with a concentration of order 10${}^{18}$ cm${}^{-3}$. Finally, the device was put on a heater and annealed at about 550 °C in a vacuum chamber evacuated under $1\times 10^{-3}$ Torr, and a 400 V${}_{\mathrm{DC}}$ voltage was applied between the comb electrodes to give an electret potential; consequently, a negative potential was formed on the positively biased comb electrodes. As shown in the lower-right inset in Figure 3, potassium ions in the SiO${}_{2}$ film of the positively biased side are ejected, and hence negatively charged oxygen deficiencies are fixed in the SiO${}_{2}$ film, which creates a negative potential between the comb electrodes. The detail of the electret procedure and its charging principle are reported in literature [25]. After the charging procedure, the maximum surface potential measured by the surface potential meter showed 303 V.

**Figure 4:**
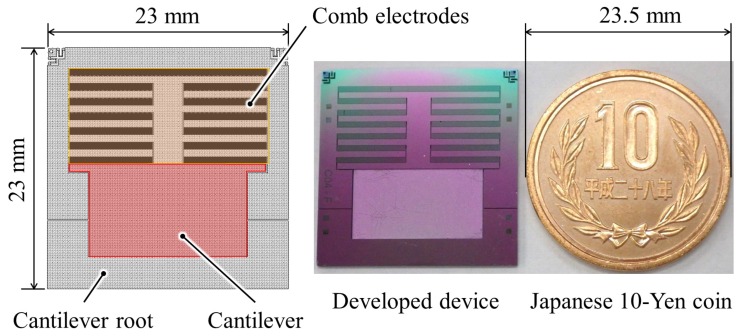
A photograph of fabricated device. Device size is 23 mm × 23 mm.

Figure 4 shows a photograph of the fabricated device. In total, 1283 pairs of the comb electrodes were formed in a fish-bone-like silicon structure that was placed at the tip of the cantilever structure. Finally, we attached a tungsten block on the backside of the fish-bone structure to weight the proof mass.

## 4. Device Performance as a Vibration Power Generator

**Figure 5:**
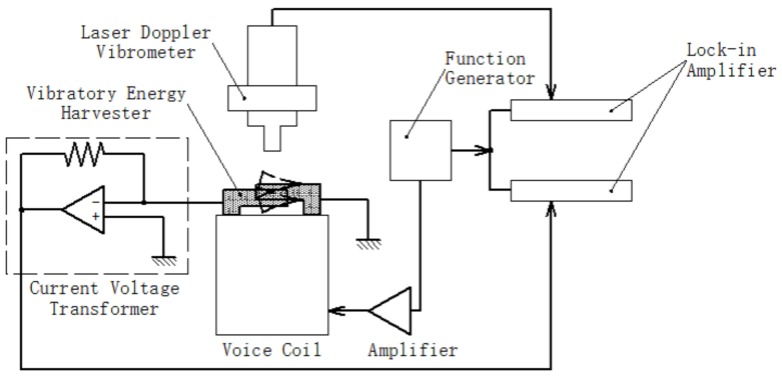
A schematic of the measurement setup to evaluate the fabricated vibration power generator.

The measurement system to examine the device performance as a vibration power generator is illustrated in Figure 5. The developed device was set on a voice-coil vibrator, and its acceleration was measured by a laser Doppler velocimeter. A load resister was connected to one of the comb electrodes, and the current flowing through the load was measured by a current–voltage transformer composed of an operational amplifier. We employed two lock-in amplifiers to take the vibration and the current signals, synchronized with a function generator that controlled the voice-coil vibration via a current boosting amplifier.

**Figure 6:**
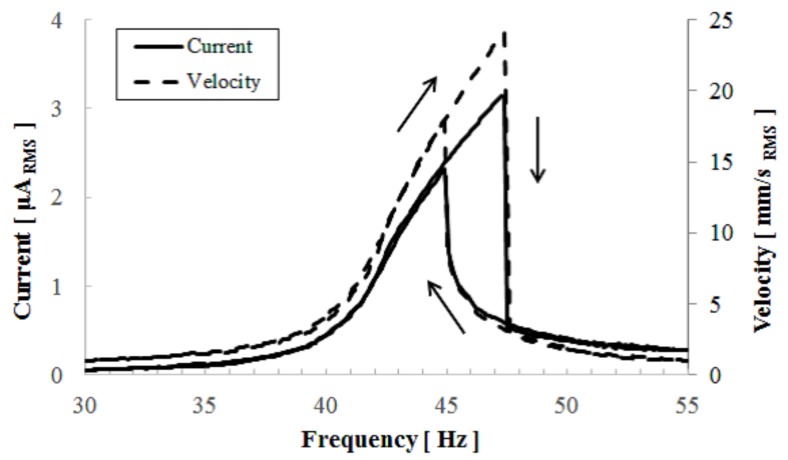
Measurement results of the short-circuit current and the vibration velocity of the cantilever as a function of external vibration frequency.

First, we attached a 14.4 g proof mass onto the developed device and measured the output short-circuit current and vibration velocity as a function of the vibration frequency. We scanned the vibration frequency in the upward and downward directions with a 0.1 Hz step in order to examine the linearity of the device response. Figure 6 shows the frequency dependency of the short-circuit current obtained by an current-to-voltage converter, in which an imaginary short condition, that is, e=0 in Equation (9), was maintained during measurements. Therefore we can examine only the first term of Equation (9). The output current gradually increased in the upward sweep and then suddenly dropped. In the downward sweep, the output current showed a steep increase at a lower frequency than the current-dropping frequency in the upward sweep. After that, the output current curve followed the curve of the current in the upward sweep. The hardening spring characteristic were clearly observed.

As suggested by Equation (10), the effective spring constant is a function of $z_{s}$ and becomes harder than the structural spring of the cantilever when $z_{s}<z_{0}/\sqrt{2}$. Although the equation was derived by a linearization theory supposing a small vibration amplitude, the effective spring constant is modulated by the displacement of the cantilever according to Equation (10); the hardening effect is caused by the electrostatic force rather than the mechanical nonlinearity referred to as the amplitude-stiffened Duffing spring effect [26].

**Figure 7:**
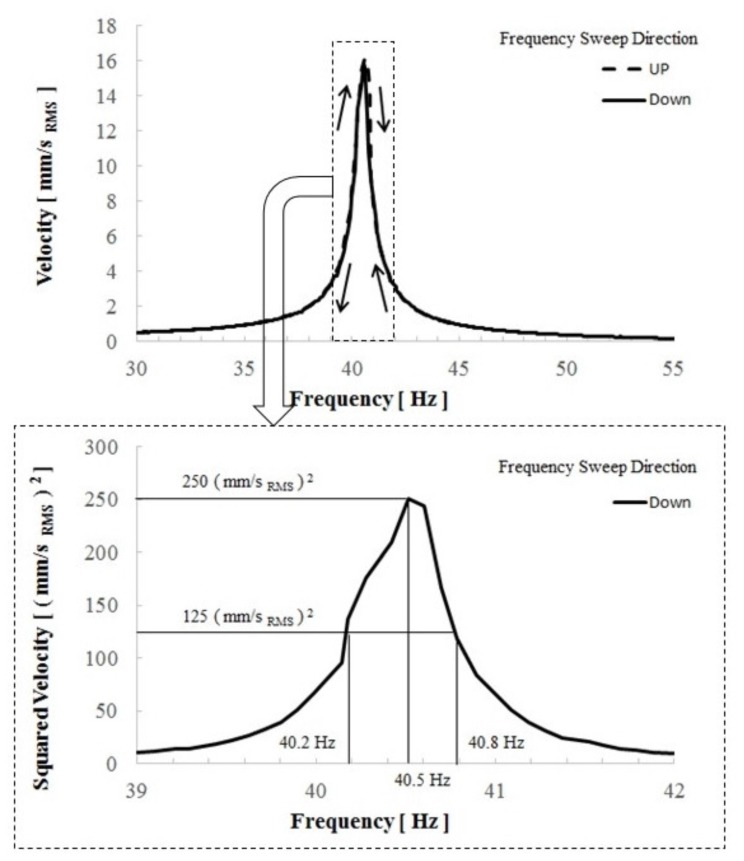
Observed vibration velocity as a function of the acceleration frequency under application of an external voltage in order to eliminate the electret potential via a protective resistance of 1 MΩ. The lower graph is the magnified view at a frequency from 39 to 42 Hz.

**Figure 8:**
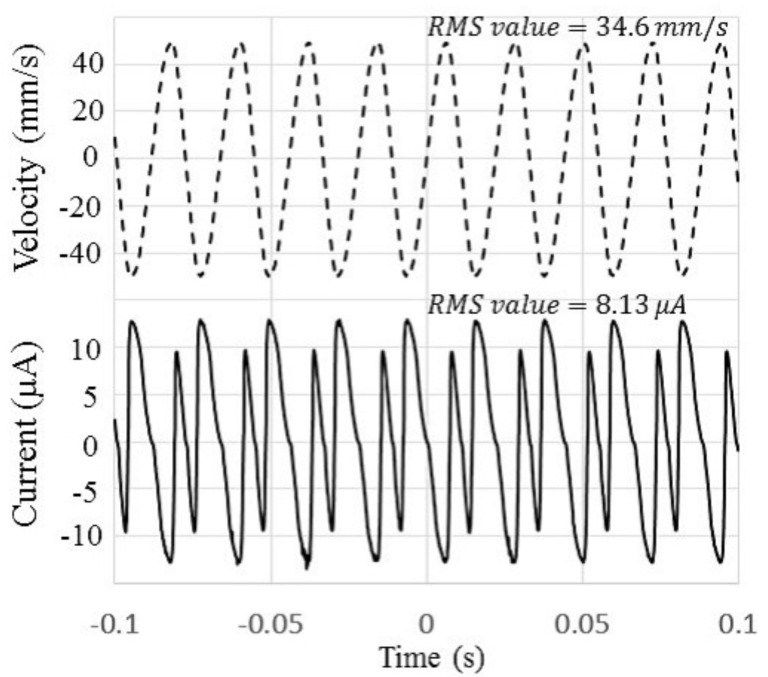
Simultaneous measurement signals of the vibration velocity and output current. Root-mean-square (RMS) values of the velocity and current are 34.6 mm/s and 8.13 μA, respectively.

Figure 7 shows the frequency response of the vibration velocity of the cantilever when an external voltage was applied to the device to eliminate the electret potential via a protective resistance of 1 MΩ. The applied excitation was under the same acceleration condition as the experiment of Figure 6. Differently to Figure 6, a linear resonance pattern was observed without the electrostatic potential, which suggests that the electrostatic force causes the nonlinearity of the vibration. As shown in Figure 6, the short-circuit current is completely proportional to the vibration velocity, and its proportional coefficient, which corresponds to the force factor in linear theory, was found to be about 1.25 × 10${}^{-4}$ C/m. Because the data were measured by using lock-in amplifiers, the force factor only reflects the synchronous current element of the output current. Figure 8 shows the obtained output current signal displayed on an oscilloscope, for which the second harmonic signal appeared. The second harmonic signal arose from the capacitance change that is represented by an even function of the displacement. Then the frequency of the electric output became double the vibration frequency. Using the measurement function installed in the oscilloscope, we found the true RMS values of the output current and velocity. From the results, a force factor of 2.34 × 10${}^{-4}$ C/m was established in the developed device.

**Figure 9:**
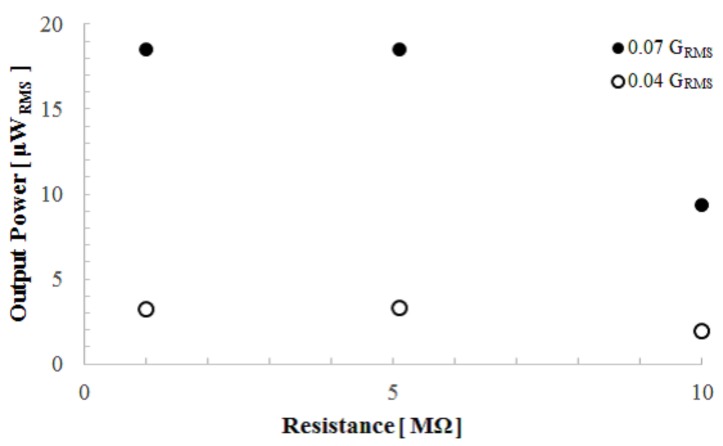
Load resistance dependence of the output power obtained by 0.04 G${}_{\mathrm{RMS}}$ and 0.07 G${}_{\mathrm{RMS}}$ external accelerations.

**Figure 10:**
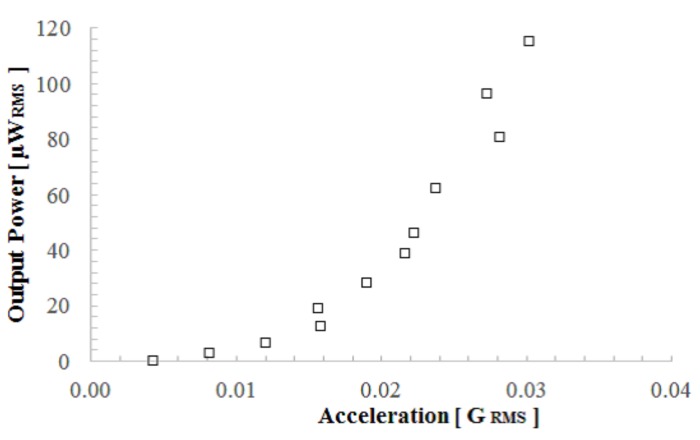
Measurement results of the output power as a function of external vibration acceleration.

Next, in order to find the optimum load for the output power measurement, we obtained the relation between the output power and the load resistance when accelerations of 0.04 G${}_{\mathrm{RMS}}$ and 0.07 G${}_{\mathrm{RMS}}$ were applied. As shown in Figure 9, the output power did not change greatly between 1 and 5 MΩ. Therefore, we measured the output power as a function of acceleration through a 1 MΩ load resistance. Figure 10 shows the experimental results of the output power that was calculated to be $1\times 10^{6}i^{2}$. Because the device showed nonlinear characteristics, as shown in Figure 6, we used the frequency at which the device delivered maximum power. The acceleration value was calculated from the vibration velocity of the voice coil by a=ωv, where ω is the angular velocity. As shown in Figure 10, over 100 μW${}_{\mathrm{RMS}}$ of electrical power was demonstrated at an acceleration of 0.03 G${}_{\mathrm{RMS}}$.

From the linear resonance graph shown in Figure 7, we found that the quality factor value was about 65 under the 1 MΩ load condition. Therefore, the intrinsic mechanical quality value was presumed to be twice as large as the obtained value, that is, a *Q* value of about 130. Furthermore, the capacitance value between the comb electrodes was measured to be 632 pF, including the parasitic capacitance, and the power efficiency defined by Equation (13) was calculated as

(15) $$\begin{array}{ccc} \eta & = & \frac{1}{1+{\left(\frac{k^{\prime }C_{0}}{QA^{2}}\right)}^{2}}\approx \frac{1}{1+{\left(\frac{m_{p}\omega_{0}^{2}C_{0}}{QA^{2}}\right)}^{2}}\\ & = & \frac{1}{1+{\left(\frac{\left(14.4\times 10^{-3}\right)\times {\left(2\pi \times 45\right)}^{2}\times \left(632\times 10^{-12}\right)}{130\times {\left(2.34\times 10^{-4}\right)}^{2}}\right)}^{2}}\\ & \approx & 0.990 \end{array}$$

where $m_{p}$ is the weight of the proof mass.

From Equation (13), $P_{max}$ is estimated to be about 143 μW${}_{\mathrm{RMS}}$ when the acceleration is 0.03 G${}_{\mathrm{RMS}}$, while the experimental output was 115.5 μW${}_{\mathrm{RMS}}$, therefore giving a conversion efficiency of η = 80.7%. The comparison of the published vibration energy harvesters under the same definition of the effectiveness was summarized in the review [9], in which the maximum efficiency of the electrostatic device was 17.9%. Despite the review’s early publishing year, we could not find another value reported since then. Although our obtained value does not completely agree with the theoretical value, we conclude that the developed device has established a very high power efficiency. Because the power efficiency depends on the force factor to the fourth-power, this high efficiency is most likely attributed to the large force factor produced by the potassium ion electrets.

## 5. Charging Experiment by an Existing Environmental Vibration

**Figure 11:**
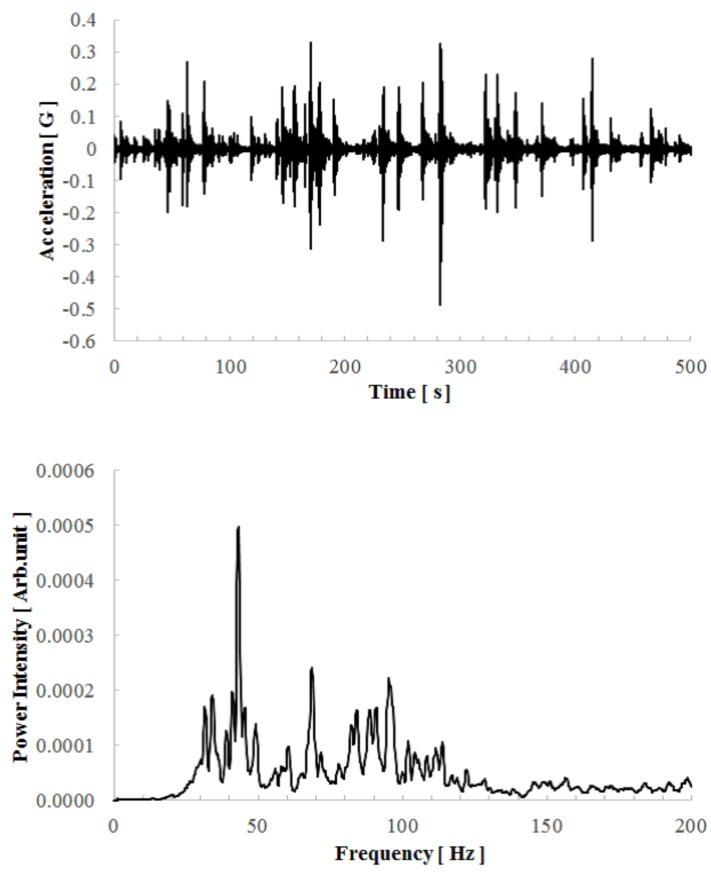
(**a**) Measured time-evolution acceleration wave taken on the viaduct of a highway; and (**b**) its power spectrum.

We conducted an electric charging experiment by using an existing environmental acceleration taken on the viaduct of a highway. The obtained acceleration wave and its fast Fourier transform (FFT) data are shown in Figure 11.

The time length of the measured data was 500 s, and the impulsive waves with different intensities were observed randomly corresponding to oncoming motor vehicles. The maximum intensity of the acceleration was about 0.5 G, and its RMS value (deviation of the acceleration) for 500 s was about 0.03 G${}_{\mathrm{RMS}}$. The FFT shows that the vibration power was distributed between 30 and 120 Hz, and the dominant frequency of that point was 43.5 Hz. We used a 44 μF capacitor as storage with a full-wave rectifier circuit composed of four silicon Schottky diodes.

**Figure 12:**
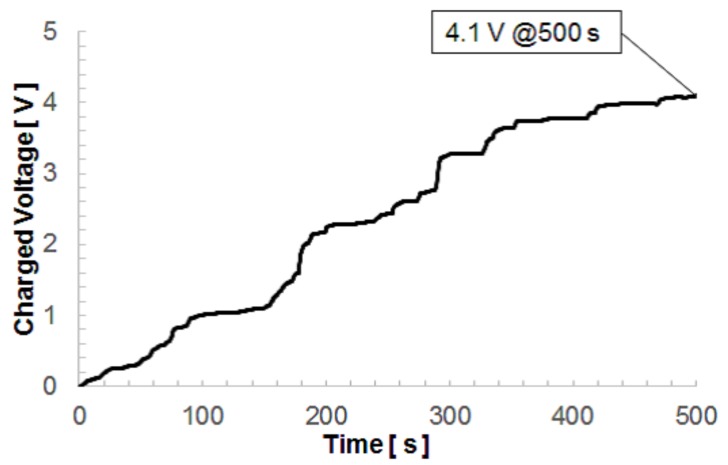
Charging voltage increment to the 44 μF capacitance obtained by the acceleration of the viaduct of a highway.

**Figure 13:**
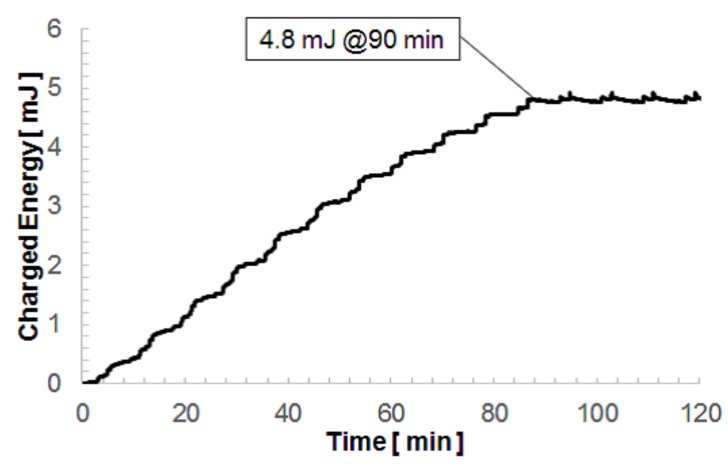
Voltage built up in a 44 μF capacitor charged by the developed energy harvester driven by the highway viaduct vibrations.

Figure 12 shows the time evolution of the charging voltage of the capacitance obtained by the developed power generator when the environmental acceleration data was applied via the voice coil. The charging voltage reached 4.09 V, corresponding to a charging energy of 368 μJ, after 500 s. We continued the charging experiment by applying the 500 s acceleration repeatedly, and the charging voltage was finally saturated at 14.7 V after 90 min. This corresponded to a charging energy of 4.8 mJ, which is large enough to drive a wireless sensor based on Zigbee technology. Figure 13 shows the time evolution of the charging energy. The charged energy in a one-cycle length time was almost the same despite that the charging voltage level was different. As demonstrated here, the developed device was found to retrieve practical electric power available from small environmental vibrations. The output voltage needs to exceed the threshold voltage of the rectifier diodes, and the output current needs to be large in order to charge the capacitor quickly. The electrostatic vibration power generator with a high force factor is thus suitable for the charging task of real environmental vibrations with small accelerations.

## 6. Conclusions

We have developed a MEMS vibrational power generator by combining semiconductor microfabrication technology and the potassium ion electret technique. The device is made of a SOI wafer with a 300 μm thick device layer, and it is composed of a cantilever structure with comb-shaped electrodes. We modeled the displacement dependency of the comb electrode capacitance by using a Gaussian function form, and derived linear motion equations as well as the force factor and the effective spring constant of the device. The developed device showed a force factor of 2.34 × 10${}^{-4}$ C/m, and more than 100 μW${}_{\mathrm{RMS}}$ of output power was obtained when sinusoidal vibrations of 0.03 G${}_{\mathrm{RMS}}$ were applied. A charging experiment using existing environmental accelerations was conducted to obtain electric power sufficient to drive a wireless sensor. This result will enable battery-free sensor network systems for the health monitoring of infrastructures.

## Figures and Tables

**Table 1:** The designed device parameters.

Parameters	Value	Unite
Spring constant, $k_{z}$	1200	N/m
Number of comb electrode pairs, *n*	1283	–
Comb electrode length, $l_{comb}$	650	μm
Comb electrode width, *w*	30	μm
Comb electrode thickness, *b*	300	μm
Comb electrode overlap, $X_{0}$	600	μm
Comb electrode gap, *d*	14	μm
Cantilever thickness, *h*	100	μm
Cantilever length, $l_{cant}$	8.82	mm
